# Cervical HPV infection and neoplasia in a large population-based prospective study: the Manchester cohort

**DOI:** 10.1038/sj.bjc.6602049

**Published:** 2004-08-03

**Authors:** J Peto, C Gilham, J Deacon, C Taylor, C Evans, W Binns, M Haywood, N Elanko, D Coleman, R Yule, M Desai

**Affiliations:** 1 Department of Epidemiology and Population Health, London School of Hygiene and Tropical Medicine, Keppel Street, London WC1E 7HT, UK; 2 Cancer Research UK Epidemiology & Genetics Unit, Institute of Cancer Research, Brookes Lawley Building, 15 Cotswold Road, Sutton SM2 5NG, UK; 3 Cervical Screening QA Reference Centre, Liverpool Women's Hospital, Liverpool L8 7SS, UK; 4 Rheumatology Section, Eric Bywaters Centre, Imperial College, London W12 0NN, UK; 5 Medical Genetics Unit, St George's Hospital Medical School, London SW17 0RE, UK; 6 Department of Histopathology and Cytology, Faculty of Medicine, Imperial College Hammersmith Hospital Campus, London W12 0NN, UK; 7 Manchester Cytology Centre, Manchester Royal Infirmary, Manchester, M13 9WW, UK

**Keywords:** cervical neoplasia, CIN3, HPV, cervical screening, cohort study

## Abstract

Cytology and histology records and cervical samples for HPV assay were obtained from a prospective cohort of 49 655 women attending clinics for routine cervical cytology in or near Manchester between 1988 and 1993. The women were followed up for cytological abnormality and neoplasia through the cytology laboratory's records. HPV at entry was assayed in an age- and period-stratified random sample of 7278 women and in prevalent and incident CIN3 cases. The prevalence of newly diagnosed CIN3 increased with time since last normal smear, indicating that most cases persist for several years. CIN3 prevalence did not increase further for screening intervals exceeding 5 years, however, suggesting that CIN3 eventually regresses cytologically. CIN2 prevalence increased less steeply with screening interval, while the prevalence of lesser abnormality was almost independent of screening interval. The prevalence of oncogenic HPV at entry declined from 19% among women aged under 25 to less than 3% at age 40 or above. Oncogenic HPV infection was strongly predictive of subsequent CIN3 (OR 17.2, 95% CI 10.4–28.4), but only weakly related to CIN2 (OR 2.3, 95% CI 0.5–10.7) and lesser abnormality (OR 1.4, 95% CI 0.8–2.5). At current incidence rates, the lifetime risk of developing CIN3 will be 9% in this population. The cumulative risk of CIN3 diagnosis among cytologically normal women with oncogenic HPV detected at entry was 28% (CI 18–43%) after 14 years. Persistence of oncogenic HPV may be more sensitive and specific than cytology for early detection of CIN3 and invasive cancer.

Population-based prospective data on cervical neoplasia rates in relation to age, screening interval and history of type-specific HPV infection are still limited. Few cohorts are representative of regularly screened women in the general population, and those using the Bethesda system under which the high-grade category (HSIL) includes both CIN2 and CIN3 (Solomon *et al*, 2002) have often not analysed CIN2 and CIN3 separately. There is a strong and consistent association of genital HPV infection with cervical intraepithelial neoplasia (CIN) and cancer. CIN3 is almost always preceded by persistently detectable oncogenic HPV (Nobbenhuis *et al*, 1999), and HPV DNA was present in virtually all (99.7%) of a large sample of cervical cancers obtained from populations worldwide (Walboomers *et al*, 1999). Uninfected women, including those with abnormal cytology, are thus at negligible risk of invasive cancer. Follow-up studies indicate that most HPV infections are transient (Hildesheim *et al*, 1994; Remmink *et al*, 1995; Ho *et al*, 1998; Liaw *et al*, 1999; Nobbenhuis *et al*, 1999), but women with persistent HPV are at high risk of developing CIN3 (Nobbenhuis *et al*, 1999, 2001). HPV testing could thus be superior to cytology in routine cervical screening (Nobbenhuis *et al*, 1999; Cuzick *et al*, 2003).

This report describes the relationship between HPV detection at entry and cytological and histological follow-up based on routine laboratory records in a prospective population-based cohort of 61 564 women recruited between 1987 and 1993. Each woman contributed at least one cervical sample for HPV analysis at a routine cervical smear test. Since the national cervical screening programme was launched in 1988, all British women aged 20–64 have been invited for cervical screening every 3–5 years, according to regional policy. Many women were therefore being screened for the first time. The cohort, in which 86% of screening intervals were over 2 years, is contemporary with the Portland cohort of over 20 000 American women in a health care plan with a policy of annual screening (Liaw *et al*, 1999; Sherman *et al*, 2003). The resulting differences in outcome are informative in relation to natural history as well as screening policy.

## METHODS

### Recruitment of the cohort

Between 1987 and 1993, in collaboration with over 100 general practitioners and screening clinics in the Greater Manchester area who used the Christie Hospital cytology laboratory (now the Manchester Cytology Centre sited at Manchester Royal Infirmary), 78 062 cervical cell samples were collected from 61 564 women attending for routine screening. There was no age restriction. Participating practices and clinics covered a wide area in and around the city of Manchester, and offered screening either in the context of well-woman clinics or in association with family-planning services.

The study was approved by the local ethics committee. Verbal informed consent obtained when the smear was taken was deemed appropriate, as the study database was anonymised, and the clinical significance of HPV infection was not then known. HPV assays were performed after recruitment had ended, and no HPV results were reported either to the cytology laboratory or to the women.

At recruitment, a cervical smear was obtained from each woman, usually using an Ayre spatula. A second scrape was then taken using the same spatula and the tip, together with adherent cervical cells, was broken off into a sterile container containing 10 ml of storage buffer (0.9% PBS (pH 7.2) +0.1% SDS). Spatula samples were sent together with the corresponding smears and pathology request forms to the Christie Hospital Cytology Department. Smears that were accompanied by a spatula were flagged on receipt on the laboratory database. The name, address and date of birth are routinely used by the cytology laboratory to match new smears against a woman's previous screening record. Spatulas were vortexed, and the cell suspensions transferred to freezer tubes, which were stored at −30°C. Samples taken before July 1988 were centrifuged and only the pellet was stored. These pelleted samples were deemed unsatisfactory for HPV assay, so all analyses are restricted to the 54 060 women who provided a sample on or after 1st July 1988.

### Structure of the study database

As a sampling frame for studies nested within the cohort, the 78 062 spatulas were stratified according to the 12-month period in which they were taken and 5-year age groups. To avoid the cost of testing all spatulas for HPV DNA, we used this sampling frame to select an age- and period-stratified random sample for HPV assay. Entry spatulas for CIN3 diagnoses notified up to June 1998 were also assayed.

Smear and histology results in the study database were updated at 6-monthly intervals during recruitment from laboratory records. Matching was on laboratory number, so named data were not required. The database was updated in the same way after recruitment had ended, and the present report includes cytology results for all smears recorded up to the end of January 1996 and histology results for biopsies taken up to the end of December 1998. (Follow-up is incomplete for symptomatic cancers, which would not necessarily be linked to the laboratory records.) In addition, screening histories of 232 women in the HPV-tested random sample who were cytologically normal but were infected with an oncogenic HPV type at entry were updated by matching against the laboratory's current database to extend their follow-up to the end of June 2002 for cytology and to the end of March 2004 for histology.

### Detection of HPV DNA

PCR analysis followed recommended anticontamination procedures (Bauer *et al*, 1992). Spatula samples were thawed at room temperature and resuspended. The cell pellets obtained from centrifugation of 250 *μ*l suspension were washed in normal saline to remove the collection buffer, then resuspended and digested with 200 *μ*g ml^−1^ proteinase K by incubation at 55°C for 1 h in 25 *μ*l 50 mM Tris-HCl buffer (pH 8.5) containing 1 mM EDTA (TE buffer), 1% (v v^−1^) Tween-20. The protease was inactivated by incubation at 95°C for 10 min and the crude digests were made up to 250 *μ*l in TE buffer. Aliquots, 5 *μ*l, of the digests were then used for HPV L1 consensus PCR amplification in 100 *μ*l reaction volume using the MY09/MY11 primers (Manos *et al*, 1989; Bauer *et al*, 1992). A 286 bp human *β*-globin fragment was amplified simultaneously in all samples to act as an internal PCR control. PCR-negative controls, and HPV-positive (SiHa cells) and negative controls were run in each experiment. Overall, 89% of all samples assayed gave a satisfactory (i.e. *β*-globin positive) HPV result. Aliquots, 10 *μ*l, of PCR product were run on agarose gel, vacuum blotted onto nylon membranes and immobilised by UV crosslinking. Membranes were hybridised with a *β*-globin oligonucleotide probe, then with a generic HPV probe in order to determine HPV positivity. Biotin-labelled probes were used and positive hybridisation was detected using enhanced chemoluminescence. Positive samples were dot blotted onto new membranes and hybridised with a series of biotinylated type-specific probes including 6/11/42 (mixed), 16, 18, 26, 31, 33, 35, 39, 40, 45, 51, 52, 53, 54, 55, 56, 57, 58, 59, 66, 68, 73, 82, 83 and 84. Samples positive with the generic probe but negative on all dot blots were considered positive but untyped.

## STATISTICAL METHODS

### Definition of diagnosis dates

There is no entirely satisfactory definition of the date of diagnosis of CIN3. The final phase of persistent cytological abnormality is frequently interrupted by a normal smear but may also be preceded by an isolated abnormal smear several years earlier. The date of histological diagnosis is not appropriate, as more than a year may elapse between the first of a series of abnormal smears and eventual histological confirmation, and this interval is determined as much by referral practice as by the rate of progression. As a practicable compromise, we have defined the date of CIN3 diagnosis in all analyses as the date of the first abnormal smear in the 2 years preceding histological confirmation of CIN3. Dates of CIN2 or cancer diagnosis were defined in the same way. Two cases of CIN3 which were diagnosed histologically between entry to the study and the following smear date, both in the third year of follow-up, in women with no recorded abnormal smear were ignored. Smears classified as cytologically inadequate were ignored in all analyses, but those that were infected but adequate were classified as normal. Abnormal smear results not followed within 2 years by histological diagnosis of CIN2 or CIN3 were classified as ‘lesser abnormality’. All adequate smears were thus classified as cytologically normal, lesser abnormality, CIN2, CIN3 or cancer. CIN3 and cancer were combined in most analyses.

### Definition of the cohorts

**Figure 1:**
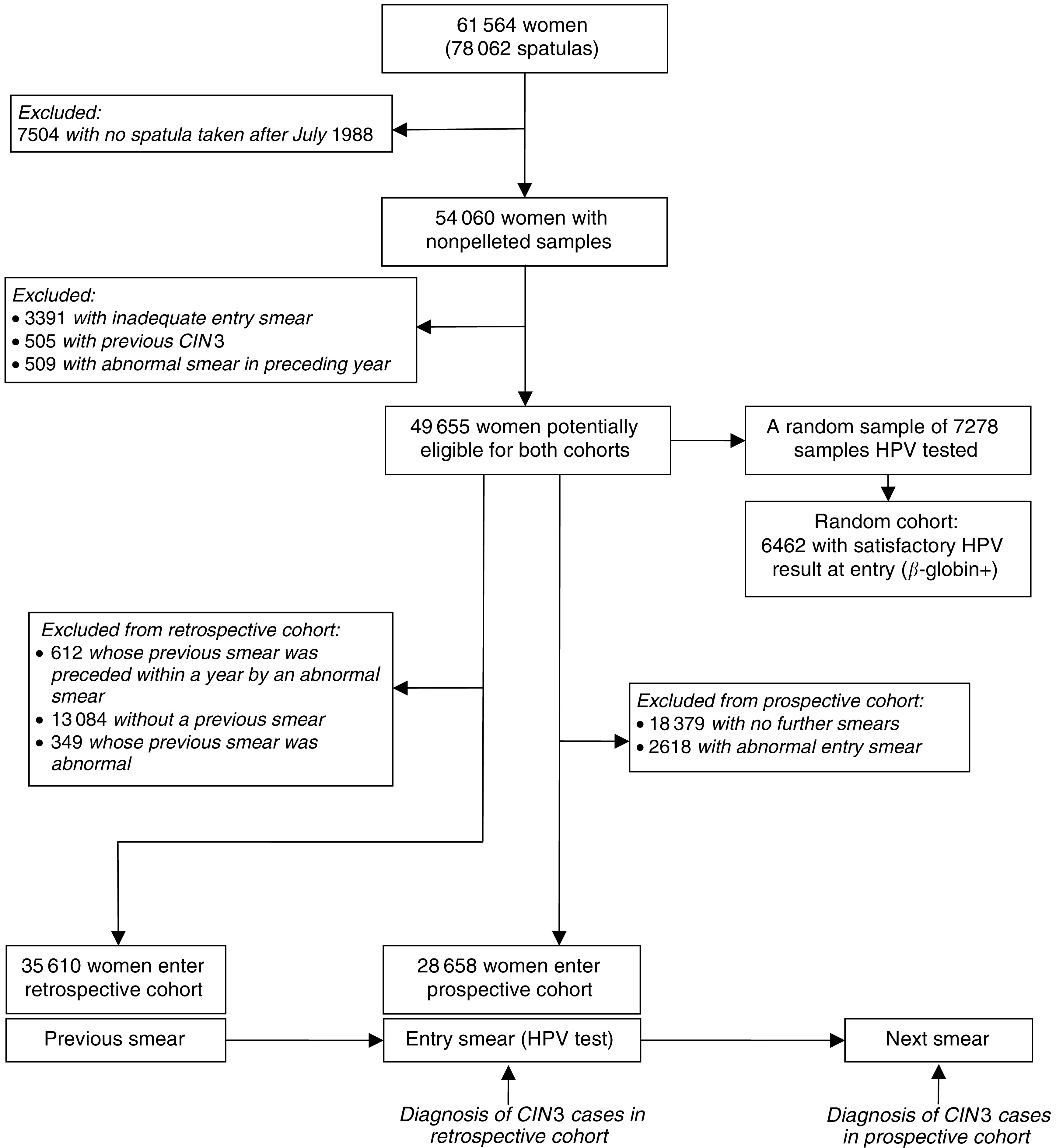
Definition of the cohorts.

The present report is based on 54 060 women who entered the study on or after 1 July 1988 (Figure 1

). The first cytologically adequate routine smear (defined as a smear not preceded by an abnormal smear within the previous year) at which a spatula sample for HPV assay was also taken defined a woman's entry date. Women were ineligible if their entry smear was inadequate (*n*=3391), they had a record of an abnormal smear within the previous year (*n*=509), or they had ever had a diagnosis of CIN3 (*n*=505). All analyses are based on the remaining 49 655 women from whom a sample for HPV assay was collected at a routine smear test that gave an adequate cytological result.

The data were analysed as two cohort studies (Figure 1). The retrospective cohort, which comprised 35 610 women whose previous smear was cytologically normal and routine (i.e. not preceded within a year by an abnormal smear), was completely followed up from the previous smear date to the entry smear date. Women with an abnormal entry smear at which CIN2 or CIN3 was diagnosed were the incident cases in the retrospective cohort. The prospective cohort, comprising 28 658 women whose entry smear was normal and who had a subsequent cytologically adequate smear, were followed from the entry smear to the following smear. Women with no record of a previous smear were included. HPV testing of entry smears was restricted to women who had at least one previous cytologically adequate smear recorded on the database. Entry spatulas for 7278 of these women were included in the age-stratified random sample of all spatulas. These 7278 spatulas were assayed for HPV, and a satisfactory (*β*-globin positive) result was obtained for 6462 (89%). These 6462 women are referred to as the random cohort.

The entry smear in the retrospective cohort and the next smear in the prospective cohort were categorised by 5-year age strata and time since the preceding normal smear for analysis by unconditional logistic regression of the odds ratios for CIN3, CIN2 and lesser abnormality. The underlying incidence of CIN3, estimated by dividing the number of cases by the woman-years of follow-up since the last smear within each age and interval stratum, was analysed by Poisson regression. HPV analysis was performed systematically on all entry spatulas (i) for the 7278 eligible women in the age-stratified random sample (ii) for all CIN3 cases diagnosed at entry and (iii) for subsequent CIN3 cases notified within the study period (i.e. with histology before the end of 1993). HPV status at entry was determined for 87% (314 out of 361) of prevalent CIN3 cases diagnosed at entry and for 41% (84 of 204) of incident cases diagnosed at the next routine smear. Logistic regression analysis was used within this nested case–control study to determine the effect of HPV infection in a normal smear on the risk of cytological abnormality at the following smear. Confidence intervals (95%) are shown for parameter estimates and significance levels are two-sided. The cumulative risk for diagnosis of CIN3 or cancer was calculated by the Kaplan–Meier method, defining diagnosis date as described above and censoring at the latest recorded smear date.

## RESULTS

### Age-specific prevalence of HPV, cytological abnormality, CIN3 and cancer at entry

Table 1

**Table 1:** Prevalence of cytological abnormality and neoplasia at entry to the study

	**Result at entry smear**													
	**CIN3 or cancer^a^**				**CIN2**				**Lesser abnormality**				**All women**	
	**No previous smears**		**Previous smears**		**No previous smears**		**Previous smears**		**No previous smears**		**Previous smears**		**No previous smears**	**Previous smears**
**Age at entry smear**	**No.**	**%**	**No.**	**%**	**No.**	**%**	**No.**	**%**	**No.**	**%**	**No.**	**%**		
15–19	5 (0)	0.2	3 (0)	0.3	4	0.1	5	0.5	160	5.2	65	5.9	3106	1099
20–24	17 (1)	0.4	28 (0)	0.5	16	0.4	33	0.6	270	6.5	353	6.3	4164	5572
25–29	16 (1)	0.8	79 (3)	1.3	17	0.9	29	0.5	112	5.8	345	5.6	1924	6179
30–34	9 (1)	1.1	74 (8)	1.5	2	0.3	28	0.6	33	4.1	201	4.1	803	4953
35–39	9 (1)	1.7	43 (3)	1.0	5	1.0	27	0.6	18	3.5	161	3.7	519	4365
40–44	3 (0)	0.7	29 (3)	0.7	2	0.5	17	0.4	22	5.0	148	3.6	443	4083
45–49	1 (0)	0.3	14 (1)	0.5	0	0.0	3	0.1	6	1.8	73	2.6	340	2857
50–54	1 (0)	0.2	13 (1)	0.5	0	0.0	9	0.4	10	2.3	41	1.6	435	2595
55+	9 (4)	0.7	8 (1)	0.2	0	0.0	3	0.1	11	0.8	28	0.6	1350	4868
														
All ages	70 (8)	0.5	291(20)	0.8	46	0.1	154	0.4	642	4.9	1415	3.9	13 084	36 571
														
All women	361				200				2057				49 655	

a Numbers and prevalence rates include cancer and CIN3. Cancers are also shown separately in brackets.

shows the prevalence of CIN3 or cancer, CIN2 and lesser abnormality at entry among the 49 655 eligible women. There were 28 cancers, 333 CIN3s and 200 CIN2s. Rates are similar at each age in women with and without a previous smear. Most of the 13 084 previously unscreened women were aged either under 25 (56%) or 55 or over (10%). Table 2

**Table 2:** Randomly selected cohort: HPV prevalence by cytology result of index smear

	**Normal smear^a^**					**Any abnormality**					**Total**						
		**Any HPV**		**High risk^b^ HPV**			**Any HPV**		**High risk^b^ HPV**			**Any HPV**		**High-risk^b^ HPV**		**HPV 16**	
**Age**	**Total**	***n***	**%**	***n***	**%**	**Total**	***n***	**%**	***n***	**%**	**Total**	***n***	**%**	***n***	**%**	***n***	**%**
15–19	319	64	20.1	55	17.2	19	17	89.5	14	73.7	338	81	24.0	69	20.4	33	9.8
20–24	466	89	19.1	75	16.1	41	24	58.5	17	41.5	507	113	22.3	92	18.2	43	8.5
25–29	586	81	13.8	61	10.4	61	38	62.3	32	52.5	647	119	18.4	93	14.4	40	6.2
30–34	1191	96	8.1	53	4.5	72	35	48.6	33	45.8	1263	131	10.4	86	6.8	42	3.3
35–39	914	36	3.9	23	2.5	49	26	53.1	24	49.0	963	62	6.4	47	4.9	22	2.3
40–44	926	26	2.8	15	1.6	46	17	37.0	13	28.3	972	43	4.4	28	2.9	13	1.4
45–49	508	18	3.5	11	2.2	19	4	21.1	4	21.1	527	22	4.2	15	2.9	6	1.1
50–54	1104	32	2.9	18	1.6	27	12	44.4	11	40.7	1131	44	3.9	29	2.6	17	1.5
55–69	114	2	1.8	1	0.9	—	—	—	—	—	114	2	1.8	1	0.9	—	—
																	
Total	6128	444	7.3	312	5.1	334	173	51.8	148	44.3	6462	617	9.6	460	7.1	216	3.3

a Includes 175 with infection.

b High-risk HPV types: 16, 18, 26, 31, 33, 35, 39, 45, 51, 52, 53, 56, 58, 59, 66, 68.

Prevalence of each HR-HPV type among the 460 HR-HPV infected women in the random cohort overall, and without another HR-HPV type: HPV16: 47.0%, 39.8%. HPV18: 16.7%, 12.0%. HPV26: 0.0%, 0.0%. HPV31: 13.0%, 8.7%. HPV33: 8.5%, 6.5%. HPV35: 1.1%, 0.7%. HPV39: 2.4%, 0.9%. HPV45: 4.8%, 3.5%. HPV51: 0.7%, 0.2%. HPV52: 3.9%, 2.0%. HPV53: 7.4%, 3.3%. HPV56: 5.0%, 2.0%. HPV58: 6.3%, 3.7%. HPV59: 1.7%, 1.3%. HPV66: 0.0%, 0.0%. HPV68: 0.0%, 0.0%.

shows the age-specific prevalence of HPV infection at entry in the random cohort of 6462 HPV-typed women. The prevalence of HPV declined from 23% among women aged under 25 to less than 4% above age 40. There is a less marked trend in women whose index smear was cytologically abnormal, among whom the prevalence declines from 68% (41 out of 60) below age 25 to 36% (33 out of 92) above age 40. In all, 75% (460 out of 617) of HPV infections included a high-risk type (listed in Table 2 footnote), and 35% (216 out of 617) included HPV16. The prevalence of HPV16 was 9% (76 out of 845) below age 25, 4% (82 out of 1910) at age 25–34 and 2% (58 out of 3707) at age 35 or over. In subsequent tables, only high-risk HPV (HR-HPV) types are considered, and cancers are included with CIN3 (designated CIN3+).

Prevalences of CIN3+, CIN2 and lesser abnormality at entry to the study and concurrent HR-HPV infection rates are summarised in Table 3

**Table 3:** Cytological status, high risk HPV prevalence and CIN3 diagnosis at entry to the main cohort

	**All women (*n*=49 655)**									
			**Abnormal entry smear**						**Women with a normal entry smear and a subsequent adequate smear result (*n*=28 658)**	
	**All women**		**CIN3+**		**CIN2**		**Lesser abnormality**		**CIN3+ at next smear**	
**Age at entry**	**No. of women**	**HR HPV prevalence^a^**	**CIN3 rate**	**HR HPV prevalence^b^**	**CIN2 rate**	**HR HPV prevalence^a^**	**Lesser abn. rate**	**HR HPV prevalence^a^**	**CIN3 rate at next smear**	**HR HPV prevalence at entry^b^**
<35	27800	12.3% 340/2755	0.8% 231/27800	86.3% 170/197	0.5% 134/27800	42.9% 6/14	5.5% 1539/27800	44.9% 66/147	1.0% 154/16114	50.8% 31/61
										
35–44	9410	3.9% 75/1935	0.9% 84/9410	77.3% 58/75	0.5% 51/9410	50.0% 3/6	3.7% 349/9410	27.3% 18/66	0.6% 35/6021	44.4% 8/18
										
45+	12445	2.5% 45/1772	0.4% 46/12445	69.0% 29/42	0.1% 15/12445	50.0% 1/2	1.4% 169/12445	13.3% 4/30	0.2% 15/6523	60.0% 3/5
										
All ages	49655	7.1% 460/6462	0.7% 361/49655	81.8% 257/314	0.4% 200/49655	45.5% 10/22	4.1% 2057/49655	36.2% 88/243	0.7% 204/28658	50.0% 42/84

a Within the random cohort (from Table 2).

b HPV status at entry was determined for 314 of the 361 CIN3 cases diagnosed at entry, and for 84 of the 204 CIN3 cases diagnosed at the next smear whose entry smears were normal.

High-risk HPV types: 16, 18, 31, 33, 35, 39, 45, 51, 52, 56, 58, 59, 68.

. The prevalence of abnormality of any grade was 7% (1904 out of 27 800) below age 35, 5% (484 out of 9410) at age 35–44 and 2% (230 out of 12 445) at age 45 or over. High-grade disease (CIN2 or CIN3+) was present in 21% (561 out of 2618) of abnormal entry smears. HR-HPV was detected in the abnormal entry smear in 82% (257 out of 314) of prevalent CIN3+ cases, with a slightly higher rate in younger women. A second spatula was taken before the diagnostic biopsy in 21 of the 57 prevalent CIN3+ cases whose entry spatula was HR-HPV negative, and 13 (62%) were HR-HPV positive. The prevalence of HR-HPV at entry among cytologically normal women in whom CIN3+ was diagnosed at the next routine smear was 50% overall (42 out of 84: Table 3, right-hand column). This prevalence was similar in smears taken less than 3 years before CIN3 diagnosis (51%:20 out of 39) or 3 or more years earlier (49%:22 out of 45).

Prevalence rates at the next smear for CIN3+, CIN2 and lesser abnormality in the combined prospective and retrospective cohorts are shown in Table 4

**Table 4:** Prevalence of CIN3+, CIN2 and lesser abnormality by interval since last normal smear and age

	**CIN3+**		**CIN2**		**Lesser abnormality**		
	**Prevalence (*n*)**	**OR (95% CI)**	**Prevalence (*n*)**	**OR (95% CI)**	**Prevalence (*n*)**	**OR (95% CI)**	**Total smears (*n*)**
Total	0.70% (428)		0.38% (230)		3.82% (2324)		60836
							
*Age at smear:*							
20–24	0.69% (65)	0.84 (0.61, 1.15)	0.59% (56)	1.15 (0.79, 1.68)	6.13% (581)	1.19 (1.05, 1.35)	9471
25–29	0.96% (107)	1.00	0.57% (63)	1.00	5.29% (587)	1.00	11093
30–34	1.23% (107)	1.18 (0.90, 1.55)	0.48% (42)	0.81 (0.54, 1.20)	4.10% (358)	0.76 (0.66, 0.87)	8729
35–39	0.78% (57)	0.71 (0.51, 0.98)	0.42% (31)	0.68 (0.44, 1.05)	3.80% (279)	0.69 (0.60, 0.80)	7334
40–44	0.67% (45)	0.58 (0.40, 0.82)	0.31% (21)	0.48 (0.29, 0.80)	3.31% (222)	0.59 (0.51, 0.70)	6708
45–49	0.37% (19)	0.30 (0.19, 0.50)	0.08% (4)	0.17 (0.04, 0.32)	3.18% (164)	0.56 (0.47, 0.67)	5156
50–54	0.28% (12)	0.23 (0.13, 0.42)	0.24% (10)	0.35 (0.18, 0.68)	1.65% (70)	0.29 (0.22, 0.37)	4234
55+	0.20% (16)	0.15 (0.09, 0.26)	0.04% (3)	0.05 (0.02, 0.16)	0.78% (63)	0.13 (0.10, 0.17)	8111
							
*Screening interval:*							
<1 year	0.16% (3)	0.16 (0.05, 0.49)	0.47% (9)	0.80 (0.40, 1.62)	4.89% (93)	1.03 (0.82, 1.29)	1903
1–1.9 years	0.40% (27)	0.41 (0.27, 0.63)	0.31% (21)	0.56 (0.34, 0.92)	4.15% (279)	0.90 (0.78, 1.04)	6717
2–2.9 years	0.67% (90)	0.85 (0.65, 1.10)	0.35% (48)	0.82 (0.57, 1.18)	4.39% (594)	1.15 (1.03, 1.29)	13533
3–3.9 years	0.71% (150)	1.00	0.38% (80)	1.00	3.45% (731)	1.00	21181
4–4.9 years	1.01% (72)	1.53 (1.15, 2.03)	0.41% (29)	1.19 (0.77, 1.82)	4.15% (295)	1.30 (1.13, 1.49)	7100
5–9.9 years	0.83% (86)	1.43 (1.09, 1.89)	0.41% (43)	1.49 (1.01, 2.19)	3.19% (332)	1.18 (1.03, 1.35)	10402
							
*Previous abnormality:*							
No	0.65% (292)	1.00	0.33% (145)	1.00	3.39% (1511)	1.00	44611
Yes	1.42% (36)	3.02 (2.11, 4.32)	0.75% (19)	2.84 (1.73, 4.66)	6.19% (157)	2.00 (1.68, 2.38)	2536
No previous smear	0.73% (100)	0.95 (0.74, 1.21)	0.48% (66)	1.04 (0.75, 1.43)	4.79% (656)	1.06 (0.95, 1.17)	13689
							
*Cohort:*							
Prospective	0.70% (197)	1.00	0.40% (112)	1.00	4.02% (1130)	1.00	28102
Retrospective	0.71% (231)	0.92 (0.76, 1.12)	0.36% (118)	0.83 (0.64, 1.08)	3.65% (1194)	0.88 (0.81, 0.96)	32734

Odds ratios were estimated by multivariate logistic regression analysis, fitting all variables shown.

by age, interval since the preceding normal smear and history of earlier cytological abnormality. Women whose next smear was taken below age 20 (*n*=1611) or after an interval of 10 or more years (*n*=1821) are excluded in Table 4. ORs were estimated by multivariate unconditional logistic regression analysis of the categorical variables shown in Table 4. Separate analyses of the retrospective and prospective data (not shown) gave similar results. The cohort indicator variable included in the combined analysis suggests a slightly lower prevalence of all three grades of abnormality in the retrospective cohort, although the difference was statistically significant only for lesser abnormality (retrospective: prospective OR 0.88, CI 0.81–0.96). The prevalence of CIN3+ increased from age 20–24 to a maximum at age 30–34 and then declined at older ages. In contrast, both CIN2 and lesser abnormality were most prevalent at age 20–24 and declined steadily with increasing age.

**Figure 2:**
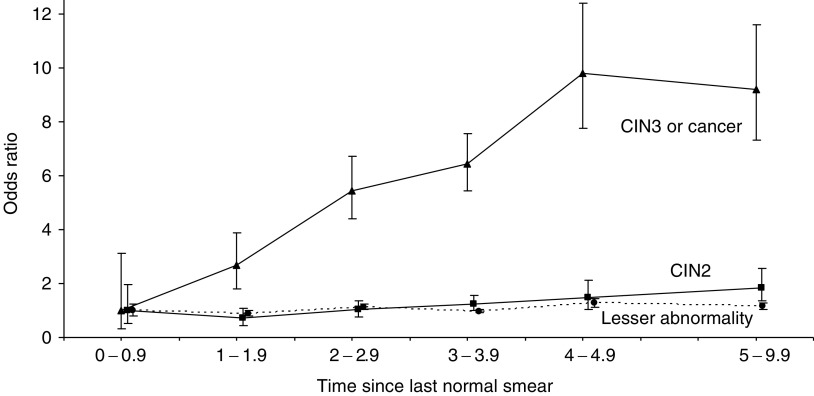
Odds ratio estimates for prevalence of CIN3+ (CIN3 or cancer), CIN2 and lesser abnormality by years since last normal smear. (Screening intervals less than 1 year taken as reference group; confidence intervals are for floating absolute risks (Easton *et al*, 1991)).

All three grades of neoplasia showed a statistically significant trend in OR with increasing screening interval, but the rate of increase was greater for CIN3+ than for CIN2, and the prevalence of lesser abnormality was almost constant (Figure 2

). Smears taken after an interval of 3–4 years were taken as the reference group (OR=1.0) in the corresponding analysis in Table 4. The prevalence of CIN3+ was 0.2% within a year of a normal smear (OR 0.16, CI 0.05–0.49) and increased by a factor of 1.49 per year (CI 1.33–1.67, *P*<0.001) for intervals of under 5 years, reaching a maximum of 1.0% (OR 1.53, CI 1.15–2.03) between 4 and 5 years. CIN3+ prevalence was 0.8% (OR 1.43, CI 1.09–1.89) for intervals between 5 and 10 years, showing no further increase. CIN2 was more common than CIN3+ initially and increased by a factor of 1.22 (CI 1.05–1.41, *P*<0.01) per year up to 5 years. The ratio of CIN2 to CIN3+ showed a significant (*P*<0.005) initial decline, from 1.5 (19 : 13) in smears taken within 18 months of a normal smear to 0.5 (211 : 415) thereafter. The OR for lesser cytological abnormality increased by only 1.05 (CI 1.00–1.10, *P*=0.03) per year since the last normal smear, from 1.03 (CI 0.82–1.29) below a year to 1.30 (CI 1.13–1.49) at 4–5 years and 1.18 (CI 1.03–1.35) beyond 5 years. The multivariate analysis shown in Table 4 was repeated for CIN2, taking women with lesser abnormality as the control group. The age distributions were similar, and the only difference between CIN2 and lesser abnormality was the slightly steeper trend in CIN2 with increasing screening interval (*P*=0.07).

The linear increase in CIN3+ prevalence with increasing screening interval up to 5 years (Figure 2; *P* trend<0.001) suggests that most new cases persist for at least 5 years. We therefore fitted the same multivariate model by Poisson regression, including the variables shown in Table 4 but replacing each smear by the number of woman-years since the last smear in the denominator. This corresponds to an analysis of the underlying incidence of CIN3+ (annual rate of development of new cases) on the assumption that new cases remain detectable at least until the next smear. The estimates for age and other variables were virtually unaltered, but relative risk (RR) estimates for different screening intervals were close to unity for intervals from under a year up to 5 years. Relative risks and CIs for intervals of <1, 1–, 2–, 3– and 4–4.9 years were 0.67 (0.21–2.11), 0.95 (0.63–1.44), 1.07 (0.82–1.39), 1.0 (reference) and 1.13 (0.85–1.50), respectively. For smears taken after 5 years or longer, however, the RR for CIN3+ incidence was less than for shorter intervals (RR 0.74, CI 0.58–0.95, *P*<0.02). The underlying age-specific incidence of CIN3 in a screened population can thus be estimated from data on screening intervals not exceeding 5 years. In Table 5

**Table 5:** Annual incidence of new cases of CIN3+ in women with a screening interval of less than 5 years following a normal smear

**Age at mid-point of interval**	**No. of CIN3+ diagnosed at next smear**	**Woman-years from last smear to next smear**	**Incidence per 1000 per year**	**Cumulative CIN3+ risk at start of interval (%)**
15–19	8	5129	1.56	0.0
20–24	50	22 487	2.22	0.8
25–29	94	23 074	4.07	1.9
30–34	72	19 623	3.67	3.9
35–39	42	17 239	2.44	5.8
40–44	23	15 065	1.53	7.0
45–49	10	11 785	0.85	7.8
50–54	7	9257	0.76	8.2
55–59	4	6861	0.58	8.6
60–64	1	5253	0.19	8.9
65–69	2	1437	1.39	9.0

, which is based on all screening intervals under 5 years in the retrospective and prospective cohorts, woman-years from the routine normal smear to the following smear and incident cases of CIN3+ are tabulated according to the woman's age at the midpoint of the screening interval. Figure 3

**Figure 3:**
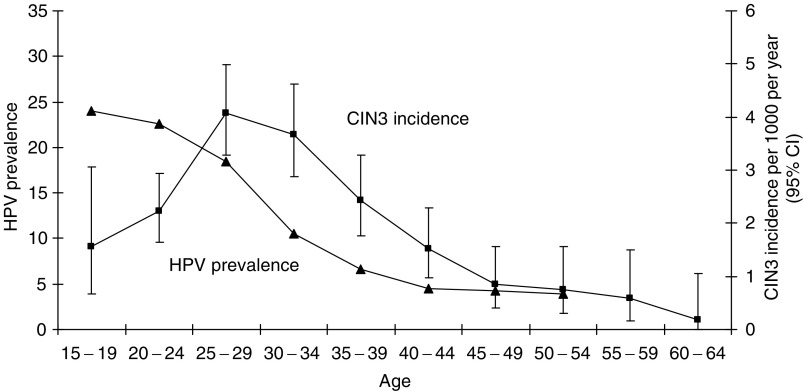
HPV prevalence and CIN3 incidence by 5-year age group.

shows the corresponding annual incidence of CIN3+ together with the age-specific prevalence of HR-HPV. The incidence of CIN3+ reaches a maximum of 0.004 per year at age 25–29 and declines steadily to less than 0.001 per year above age 45. These incidence rates imply a cumulative risk of developing CIN3+ of 7.8% by age 45 and 9.0% by age 65 (Table 5).

### Risk among cytologically normal HPV carriers

**Figure 4:**
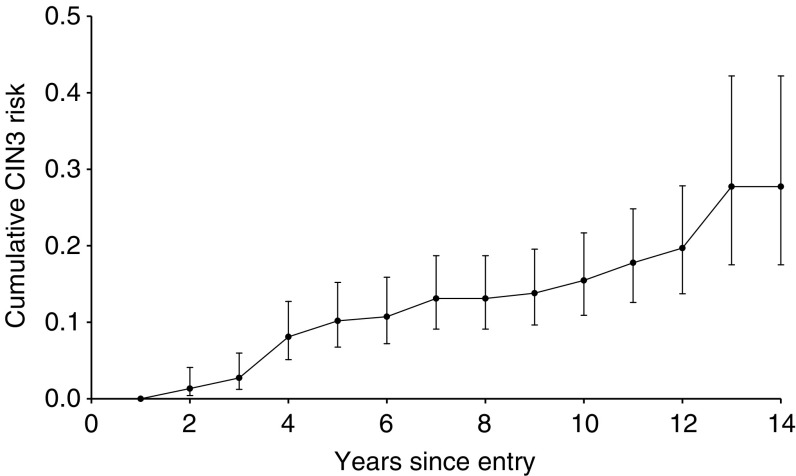
Cumulative risk of CIN3 or cancer among 232 cytologically normal women with HR-HPV infection at entry.

The random cohort included 232 cytologically normal women with HR-HPV at entry and at least one later smear who were followed cytologically for up to 14 years. In all, 15% developed histologically confirmed CIN3+ (28 CIN3s and six cancers), giving a cumulative actuarial CIN3+ diagnosis rate of 16% (CI 11–23%) after 10 years and 28% (CI 18–43%) after 14 years (Figure 4

).

The high risk of subsequent CIN3+ in women with HR-HPV infection is apparent from Table 6

**Table 6:** High-risk HPV prevalence at entry in the random cohort among women with a normal entry smear and an adequate subsequent smear

	**Next smear result**				
**Age at next smear**	**Normal**	**CIN3+**	**CIN2**	**Lesser abnormality**	**All women**
*HR*^a^ *HPV positive at entry*					
<35	144 (87.8%)	11 (6.7%)	0 (0.0%)	9 (5.5%)	164
35–44	35 (76.1%)	5 (10.9%)	2 (4.4%)	4 (8.7%)	46
45+	17 (77.3%)	4 (18.2%)	0 (0.0%)	1 (4.6%)	22
					
All ages	196 (84.5%)	20 (8.6%)	2 (0.9%)	14 (6.0%)	232
					
*HR*^a^ *HPV negative at entry*					
<35	1109 (92.9%)	12 (1.0%)	9 (0.8%)	64 (5.4%)	1194
35–44	1283 (95.1%)	6 (0.4%)	3 (0.2%)	57 (4.2%)	1349
45+	1536 (97.0%)	2 (0.1%)	2 (0.1%)	44 (2.8%)	1584
					
All ages	3928 (95.2%)	20 (0.5%)	14 (0.3%)	165 (4.0%)	4127
					
All women	4124 (94.6%)	40 (0.9%)	16 (0.4%)	179 (4.1%)	4359

a High-risk HPV types: 16, 18, 26, 31, 33, 35, 39, 45, 51, 52, 53, 56, 58, 59, 66, 68.

. Among women with HR-HPV but normal cytology at entry, the proportion whose following smear was abnormal was 16% (36 out of 232). The majority (20) of these 36 abnormal smears had underlying CIN3+, two had CIN2 and the remaining 14 had lesser abnormality. The prevalence of CIN3 increased with age, but the trend was not significant. In contrast, the CIN3 prevalence at next smear for uninfected cytologically normal women was much less and declined with increasing age, from 1.0% (12 out of 1194) below age 35 to 0.1% (two out of 1584) at age 45 or over. The relationship between initial HR-HPV infection and neoplasia at the next smear was analysed by unconditional logistic regression of the 84 CIN3+ cases whose HPV status at entry was known (Table 3) using the 4124 HPV typed women in the random cohort hose next smear was normal as controls (Table 6). The estimated OR for CIN3+ at the next smear associated with HR-HPV infection in a normal entry smear was 17.2 (CI 10.4–28.4, *P*<0.001). Analysis restricted to the 40 incident CIN3+ cases (Table 6) in the random cohort gave a similar OR estimate (OR 21.1, CI 10.4–42.9). This analysis in the random cohort was repeated with lesser cytological abnormality as the outcome event. The prevalence of HR-HPV infection in the normal entry smear was 8% (14 out of 179) among women whose follow-up smear showed lesser abnormality, and the corresponding odds ratio was 1.4 (CI 0.8–2.5). Although based on only 16 incident CIN2 cases for whom HPV at entry was determined, the corresponding analysis for women who developed CIN2 gave similar results. The prevalence of HR-HPV in their normal entry smears was 13% (two out of 16) and the CIN2 odds ratio for prior HR-HPV infection was 2.3 (CI 0.5–10.7).

### HPV type-specific risk

It has long been recognised that genital (and other) HPVs can be classified phylogenetically into groups (Van Ranst *et al*, 1992) that confer different risks of disease (Lorincz *et al*, 1992). Table 7

**Table 7:** Distribution of HPV types in CIN3s diagnosed at entry, and in HPV carriers in the random sample who did not have CIN3 at entry.

**HPV type**	**CIN3+ with HPV+ve entry smear**	**Random sample of other women HPV+ve at entry**	**OR (exact 95% CI)**
16	182 (68.4%)	165 (30.6%)	1.00
16 related^a^	46 (17.3%)	109 (20.2%)	0.38 (0.25–0.58)
18	18 (6.8%)	62 (11.5%)	0.26 (0.14–0.47)
18 related^b^	9 (3.4%)	28 (5.2%)	0.29 (0.12–0.66)
Other HR^c^	2 (0.8%)	26 (4.8%)	0.07 (0.01–0.29)
LR and NK^d^	9 (3.4%)	150 (27.8%)	0.05 (0.02–0.11)
Total	266 (100%)	540 (100%)	

a Types 31, 33, 35, 52, 58.

b Types 39, 45, 59, 68.

c Other high-risk HPV types: 26, 51, 53, 56, 66.

d Low risk or unknown HPV types, including generic probe +ve but type unidentified.

When multiple HPV types were detected, women have been categorised by their highest risk virus according to the ranking in the table.

shows the distribution of HPV types among 266 HPV-infected women with CIN3+ at entry and in the 561 carriers in the random cohort who did not have CIN3+ at entry. The risk is highest for HPV 16, which constituted 68% of HPV infections among CIN3+ patients but only 31% among unaffected carriers. The OR is three to four times lower for the 27% of CIN3+ cases infected with other oncogenic HPV types in the same class as HPV16 or the HPV18-related class. Other HPV types account for 33% of prevalent infections but only 4% of CIN3+ cases. The prevalence of CIN3+ at the following smear was thus highest for women with HPV16.

A second spatula was not obtained for most women in the cohort, and HPV status at the following routine smear could be determined for only 86 women who were cytologically normal but HR-HPV positive at entry. The proportion still infected with the same HR-HPV type at their following smear was 31% (27 out of 86). All eight women who were initially HR-HPV positive and were diagnosed with CIN3 at their following smear had the same HR-HPV type in both samples (seven HPV16, one HPV18). The same HR-HPV type was detected at entry and at the next smear in 21 (32%) of the 65 women whose next smear was taken after an interval of more than 2 years. The next smear was abnormal in 13 (62%) of these 21 women with persistent HR-HPV, including eight with CIN3+, two with CIN2 and three with lesser abnormality. HPV16 was more likely to persist than other HR-HPV types. The proportion of women with HR-HPV at entry who still had the same HPV type at their next smear was 53% (18 out of 34) for HPV16, 26% (nine out of 35) for HPV18, HPV31 or HPV33 and none of 27 for all other HR-HPV types.

## DISCUSSION

### HPV detection predicts CIN3 but not lesser abnormality

The relationship between HR-HPV infection at entry and low-grade abnormality at the next smear was weaker in this study (OR 1.4, CI 0.8–2.5) than in several other cohorts (Liaw *et al*, 1999),(Kjaer *et al*, 2002), (Schlecht *et al*, 2001). This difference is at least partly due to the longer screening intervals in Manchester, which gave most transient HPV infections and their associated lesions time to disappear. In the Portland cohort with a policy of annual screening, the prevalence of HPV infection at entry among women who subsequently developed ASCUS or LSIL declined from 60% for screening intervals of 17 months or less to 24% for intervals of 3 years or longer (Liaw *et al*, 1999). Cytologically normal women with HPV infection at entry also had a higher risk of dysplasia in smears taken after a shorter interval than in later smears in other cohorts (Schlecht *et al*, 2001; Kjaer *et al*, 2002). In addition, our lesser abnormality category included some mildly abnormal smear reports that would have been excluded by the rigorous pathological review conducted in these three cohorts. The combined prevalence of HR-HPV at diagnosis was 64% for ASCUS or worse smears in Portland (Sherman *et al*, 2003) and only 44% for all abnormal smears in our study (Table 2). Any relationship between HPV infection and later risk of low-grade lesions was further reduced in Manchester by the exclusion from our lesser abnormality category of smears that were followed within 2 years by histological diagnosis of CIN3+, most of which contained HPV.

### Sensitivity of HPV testing in primary screening

Our sample collection protocol was not designed for PCR analysis and was unsatisfactory by modern standards. *β*-Globin could be amplified in only 89% of samples, due probably to the inhibitory effect of the wooden spatula tip, which remained in the sample at room temperature for several weeks before decanting and freezing. Among those that were *β*-globin positive, however, these sampling and assay methods detected HR-HPV in 82% (257 out of 314) of the CIN3+ cases diagnosed at an abnormal entry smear (Table 3). This is similar to the average sensitivity of 84% for HSIL detection by HPV assay reported in a recent meta-analysis of published studies (Arbyn *et al*, 2004). A second spatula was taken before histological diagnosis from 21 of the 57 women with prevalent CIN3+ whose initial spatula was HR-HPV negative, and 13 (62%) were HR-HPV positive. These statistics suggest that about 95% of CIN3s were HPV positive, but this rate would presumably have been higher with the lower detection threshold of modern PCR methods. Regular HPV testing of sufficient sensitivity would thus detect HR-HPV in almost all CIN3 cases (Nobbenhuis *et al*, 1999; Cuzick *et al*, 2003). The right-hand part of Table 3 shows that the prevalence of HR-HPV at entry among cytologically normal women in whom CIN3+ was diagnosed at the next routine smear was 50% (42 out of 84). More sensitive HPV testing would thus detect the majority of CIN3+ cases several years before they become cytologically abnormal. In the Portland study the prevalence of HR-HPV at entry in cytologically normal women was 73% in those who developed CIN3+ within 45 months and 38% in those diagnosed later (Sherman *et al*, 2003). Women with incipient CIN3 usually shed HPV persistently for several years before becoming cytologically abnormal (Nobbenhuis *et al*, 1999), so cases detected by HPV screening would still be HPV positive on retesting, and would be referred for colposcopy irrespective of the rescreening interval.

The sensitivity of our HPV assay must depend on viral load, and would be lower in women with normal cytology. Using MY-Gold instead of MY-Taq polymerase increased the HR-HPV detection rate by 54% in women with normal cytology, but only by 2% in women with HSIL or cancer (Castle *et al*, 2002). Our HPV prevalence estimates (Table 2) may thus be substantially too low at each age.

### Lifetime HPV and CIN3 rates

The relationship between HPV prevalence based on a single sample and lifelong risk of infection is not known. Persistent or recurrent infection is more likely to be detected than a transient episode due to the length-biased sampling effect. The cumulative HPV rate was higher than the cross-sectional rate by a factor of 1.6 among American students sampled weekly on 10 occasions (Wheeler *et al*, 1996), and by about two-fold among Brazilian women sampled four times at 4-monthly intervals (Schlecht *et al*, 2001). The proportion of younger women in our cohort who will acquire an HR-HPV infection during their lifetime is thus likely to be at least twice the prevalence of 18% seen in women aged 20–24 (Table 2). At current rates 9% of the cohort will eventually develop CIN3 (Table 5), although this may be an underestimate, as both CIN3 and HPV rates appear to have increased over the following decade. More than 25 000 women were recruited to the ARTISTIC trial of primary HPV screening between 2001 and 2003 through many of the same clinics in and around Manchester. HR-HPV prevalence by Hybrid Capture II was 33% at age 20–29, 15% at 30–39, 9% at 40–49 and 6% at 50–69 (Kitchener *et al*, 2004), more than double the rate we observed in the same population about 12 years earlier at each age (Table 2). This large increase seems unlikely to be due entirely to differences in HPV detection sensitivity. The CIN3 incidence rate in England and Wales at age 20–24 doubled from 1.2 per thousand in 1990 to 2.4 per thousand in 2000, and the lower prevalence of cervical abnormality in the retrospective cohort compared with the prospective cohort (Table 4: OR 0.88, *P*<0.05) is also consistent with a continuing increase in HPV prevalence during the 1990s.

Transiently detectable HPV infection is generally regarded as innocuous, but our long-term follow-up of 232 women with HR-HPV at entry shows a cumulative CIN3+ diagnosis rate of 16% after 10 years (Figure 4). A lower CIN3 rate following HPV detection was seen in the Portland study, where the cumulative risk of CIN3 or cancer diagnosis among women with HR-HPV infection and normal cytology at entry was only about 6% after 10 years' follow-up. The risk in the Portland cohort was even lower among those who were cytologically abnormal (ASCUS or worse) at entry after the initial lesion had regressed or been treated (Sherman *et al*, 2003). Only one CIN3 was diagnosed in the Portland study in smears taken between 21 months and 10 years after entry among 417 HR-HPV positive women with an abnormal entry smear compared with 38 cases among 2562 women with HR-HPV infection but normal cytology at entry (OR 0.16, *P*=0.03). This reduction in risk supports the long-standing belief that even biopsy of HPV lesions may reduce the subsequent risk of CIN3 (Koss *et al*, 1963). Cervical ablation substantially reduced the risk of subsequent CIN or cancer when this was a routine procedure in cytologically normal women (Vonka *et al*, 1984). This protective effect, combined with an annual screening policy under which a substantial proportion of HR-HPV-infected women will eventually be detected with dysplasia, may account for the lower overall CIN3 risk among infected women in the Portland study. The increased CIN3 risk among women in Manchester with a history of cervical abnormality (OR 3.0: Table 4) presumably reflects their high probability of having an HR-HPV infection, and perhaps also a lower probability in Britain than in the US that their dysplasias were biopsied or destroyed.

### Cytological regression of CIN3

The CIN3 prevalence in smears taken more than 5 years after a normal smear appears to have reached a plateau at about 1% (Table 4 and Figure 2). The initial linear increase in CIN3 prevalence with increasing screening interval shows that CIN3 often remains detectable for at least 5 years, but this subsequent plateau suggests that most cases eventually regress cytologically. The rising prevalence thus represents a balance between increasing CIN3 incidence and cytological regression. The inference that most CIN3s are only transiently detectable is supported by the low prevalence of CIN3 in women aged 45 or older with no record of a previous smear, which was only 0.5% (Table 1; 11 out of 2087). A proportion of these women would have had unrecorded smears, but the CIN3 incidence rates in Table 5 suggest that 8% of women in this cohort develop CIN3 by age 45. Most earlier CIN3s must therefore have regressed cytologically by the time these unscreened women entered our study. Even in the high-risk rural population of Costa Rica, the prevalence of persistent HSIL at initial recruitment was less than 1% above age 45 (Herrero *et al*, 2000). Reversion to cytological normality in women with underlying carcinoma *in situ* was observed directly in the prospective study of Dutch women referred for colposcopy with abnormal smears, in which six of the eight women who regressed to normal cytology and colposcopy but remained HR-HPV positive had occult CIN3 (Nobbenhuis *et al*, 1999).

The underlying risk in women who choose not to be screened may differ between different populations. The CIN3 rate at entry in women with no previous smear history was similar to that in the screened population both in our cohort (Table 1) and in a Norwegian study (Forsmo *et al*, 1996), and the rate at their second smear was slightly less than in previously screened women (Table 4). A much lower CIN3 rate was observed at the second smear than at entry in a national study of inadequately screened low-income American women (Sawaya *et al*, 2003), but this may reflect confounding between screening interval and number of negative smears. Above age 30 both CIN3 and cancer were slightly less common in unscreened women in the American study (Sawaya *et al*, 2003) than in our cohort (Table 1), and CIN3 prevalence after a negative smear was similar to the rate we observed for screening intervals of under 2 years (0.35%: Table 4).

### National data linkage

The underlying age-specific incidence rate of new cases of CIN3 in Manchester shown in Figure 3 could be estimated in the same way in any population with routine screening records linked to histology records. This could now be done for the whole of Britain, as the computerised call–recall system introduced in 1988 includes details of almost all cervical smears for the whole population aged 20–64. Individual women's smear records are already linked to histological diagnoses of CIN3 in the computer systems of the cytology laboratories that provide these data. Linkage to cancer registrations and deaths would provide CIN3 incidence as well as cancer incidence and mortality rates by screening interval and age in all regularly screened women, complemented by cancer rates in the inadequately screened remainder of the population. Sasieni and Cuzick (2001) recommended that such audit should be established as a routine procedure. The files could also be linked to small area statistics such as deprivation index that can be derived from address postcodes. The files would be anonymised after linkage, so informed consent would not be needed for such analyses.

### Progression to carcinoma *in situ* and cancer

Three observations within our cohort reflect the distinction between the majority of HR-HPV infections that resolve within a year or two and the minority that persist and frequently progress to CIN3 (Nobbenhuis *et al*, 1999). First, the prevalence of CIN3 increases linearly with time since the last normal smear, at least up to 5 years, while that of lesser (low-grade) abnormality is almost independent of screening interval (Figure 2). Second, there is a very strong relationship between HR-HPV infection at entry in cytologically normal women and the risk of CIN3+ at their next smear (OR 17.2, *P*<0.001), but a much weaker and nonsignificant relationship for abnormalities other than CIN3+ (OR 2.3 for CIN2 and 1.4 for lesser abnormality). Third, the much lower incidence of CIN3 in women aged under 25 despite their higher HPV prevalence compared with those aged 25–34 (Figure 3) indicates a substantial delay between initial HPV infection and the development of carcinoma *in situ*. In a case–control study nested within the cohort (Deacon *et al*, 2000), the number of sexual partners was the main risk factor for detectable HPV infection, but progression to CIN3 diagnosis among HPV-infected women depended more on early age at first intercourse, providing further evidence that carcinoma *in situ* often originates in a much earlier HPV infection.

An increase in prevalence of CIN2 and CIN3 but not of lesser abnormality with increasing interval since the last normal smear was reported in Icelandic women, but data for CIN2 were not presented separately (Sigurdsson and Adalsteinsson, 2001). There are several epidemiological differences between CIN2 and CIN3. The CIN2 : CIN3+ ratio was 1.5 (19 : 13) in smears taken within 18 months of a normal smear and 0.5 (211 : 415) for longer intervals (*P*<0.005). CIN2 is also commoner in young women. The CIN2:CIN3+ ratio fell from 1.1 (58 : 53) below age 25 to 0.5 (142 : 308) at older ages (Table 1; *P*<0.001). Persistently detectable HPV preceding cytological abnormality is common in the development of CIN3 and cancer but not of CIN2. In a prospective cytological and colposcopic follow-up of women in the Netherlands with abnormal smears (Nobbenhuis *et al*, 1999), the proportion of incident cases that were preceded by persistent HR-HPV infection was 95% (98 out of 103) for CIN3, but only 27% (eight out of 30) for CIN2 and 10% (six out of 63) for CIN1. HR-HPV was detected in the preceding normal smear in 50% (Table 3: 42 out of 84) of CIN3s and in 13% (Table 6: two out of 16) of CIN2s in our cohort (*P*<0.01). Despite disagreement between pathologists over the classification of individual cases, these objective differences between CIN2 and CIN3 support the view that most CIN2s are biologically and clinically indistinguishable from the lower grade HPV lesions that usually regress (Syrjanen *et al*, 1992). Among women with HPV infection, these authors observed progression or recurrence in 81% of CIN3s, 24% of CIN2s and 17% of CIN1s (Syrjanen *et al*, 1992). The weak increase in CIN2 prevalence with increasing screening interval (Figure 2) confirms that some cases are persistent, perhaps because they harbour undetected carcinoma *in situ* or because they are precursors in its progression. For clinical purposes, the inclusion of CIN2 with CIN3 in the HSIL category of the Bethesda classification may therefore be justified, although a repeat smear would avoid colposcopy and biopsy in the transient majority of CIN2s (Syrjanen *et al*, 1992). Epidemiological studies in which CIN2 and CIN3 are combined are unnecessarily uninformative, however, particularly in younger women or if the screening interval is 18 months or less. Clonality, HPV integration, loss of heterozygosity and patterns of gene expression are likely to reflect different stages of carcinogenic progression (Evans and Cooper, 2004), and results for CIN2 and CIN3 should also be presented separately in cellular and molecular studies. It is perhaps unfortunate that the earlier categories of severe dysplasia and carcinoma *in situ* were superseded (Syrjanen *et al*, 1992), despite difficulties of reproducibility (Sherman, 2003).

The age-specific CIN3 incidence pattern shown in Figure 3 suggests a simple model for the relationship between CIN3 and invasive cancer. Before the introduction of the national screening programme in 1988, cervical cancer death rates in Britain rose sharply up to about age 45, then remained almost constant above age 50 within each birth cohort (Peto *et al*, 2004). The cumulative risk of developing CIN3 (Table 5) shows a similar pattern. In view of this similarity and the effectiveness of regular screening in preventing cervical cancer, it seems likely that the incidence of cervical cancer remains roughly constant at about 1% per year for the rest of their lives in women who have developed undiagnosed CIN3, and about 40% will eventually develop invasive cancer (Peto *et al*, 2004).

### Implications for screening policy

If most CIN3s eventually regress cytologically, an increasing proportion will be missed as the screening interval increases and many may not reappear until they become malignant. CIN3 regression is also relevant in choosing the age at which screening should begin. The age at which routine screening begins has recently been raised from 20 to 25 years in the UK, and it has been suggested that this should be further increased to 30 years because invasive cancer is so rare in young women (Miller, 2002). Registered CIN3 rates for England for 2000 imply that 2.9% of women are now diagnosed with histologically confirmed CIN3 by age 30, and the underlying risk must be even higher, as coverage is still incomplete. If screening began at age 30, therefore, at least 3% of women would already have had undiagnosed CIN3. Table 5 suggests that 3.9% of women in our cohort develop CIN3 by age 30. The majority of such cases may no longer be cytologically detectable at age 30, as the prevalence of CIN3 in women with no previous smear (Table 1) was only 0.8% (16 cases) at age 25–29 and 1.1% (9 cases) at age 30–34.

CIN3 in an older woman who has not been screened regularly may often be a recurrence of an earlier undiagnosed lesion. The CIN3 rate in England was increasing at all ages before 1988, but the rate among women aged over 45 has fallen progressively since the introduction of national screening. Women whose CIN3 has already regressed before their first smear and does not recur may suffer an increased risk of invasive cancer for the rest of their lives, and this long-term risk could be an important disadvantage of delaying the age at which routine screening begins (Peto *et al*, 2004).

Our results support the conclusion that the screening interval should be less than 5 years, except perhaps for older women who are HPV negative, whose risk of CIN3 is very low (Table 6). Our analysis suggests that more frequent screening will have little effect on the overall number of CIN3 cases diagnosed in a regularly screened population. More frequent screening will prevent more CIN3s by ablation of lesser lesions; but it will also detect more before they have regressed cytologically, and will therefore reduce cancer incidence. The anomalous long-term protective effect of abnormal cytology among HPV-infected women in Portland suggests that the annual cytology, routine colposcopy of ASCUS and more frequent ablative treatment still practised by most American gynaecologists (Noller *et al*, 2003) will over time lead to a reduction in CIN3 incidence. The additional reduction in invasive cancer achieved by annual rather than 3-yearly screening is likely to be small (Sawaya *et al*, 2003), however, and many lesions that are HPV-negative and hence harmless will also be treated. Sensitive HPV testing is therefore a useful adjunct to cytology (Cuzick *et al*, 1995), as mildly abnormal smears that do not contain HR-HPV can be identified and ignored (Cox *et al*, 2003). The potential superiority of HR-HPV testing over cytology for sensitive primary screening is now widely recognised. Very sensitive HPV testing will reduce still further the CIN3 or cancer risk in HPV-negative women and increase the proportion of CIN3s that are preceded by persistent HPV detection, but it is also likely to increase the number of referrals for colposcopy. Other practical questions that must be resolved before HPV testing can replace cytology are the appropriate screening interval, whether HR-HPV infection should be observed or treated immediately by cervical ablation, and whether cytology is worthwhile in addition to HPV testing.

### Participating clinics

*Manchester:* Abbey Hey Clinic, Alexandra Park Health Centre, Ancoats Community Clinic, Ancoats Hospital, Assheton Road Clinic, Atherton Clinic, Baguley Clinic, Beswick Health Centre, Bodey Medical Centre, Boothtown Clinic, Brunswick Clinic, Central Drive Clinic, Charlestown Health Centre, Child Health Centre, Clayton Health Centre, Corporation Road Clinic, Crumpsall Clinic, Derbyshire House Clinic, Gorton Combined Clinic, Harpurhey Health Centre, Hulme Clinic, Irlam Health Centre, Ladybarn Group Practice, Levenshulme Health Centre, Little Hulton Clinic, Longsight Health Centre, Monton Road Clinic, Moss Side Health Centre, Newton Heath Health Centre, Nicholas Road Health Centre, Northenden Group Practice, Northern Hospital, Partington Lane Clinic, Plant Hill Clinic, Poplar Street Clinic, Robert Darbyshire Practice, Smedley Street Clinic, St Mary's Hospital, The Palatine Centre, Varley Street Clinic, Walkden Clinic, Walmer Street Clinic, Wellwoman Clinic, Wilmslow Road Clinic, Woodhouse Park Clinic, Wythenshawe Health Care Centre. *Rochdale:* Whitworth Medical Centre. *Salford:* Bevendon Square Clinic, Higher Broughton Health Centre, Hope Hospital, Lanceburn Health Centre, Liverpool Street Clinic, Lower Broughton Clinic, Ordsall Health Centre, Langworthy Clinic. *Stockport:* Adswood Clinic, Bramhall Clinic, Brinnington Clinic, Burley House Clinic, Cheadle Heath Clinic, Hazel Grove Clinic, Heaton Norris Clinic, Maple Clinic, Marple Cottage Surgery, North Reddish Clinic, Offerton Clinic, Offerton Green Clinic, Robins Lane Medical Centre, Romiley Clinic, Romiley Health Centre, Shaw Heath Clinic, Stepping Hill Clinic, Wellwoman Clinic, Woodley Clinic, Bredbury Clinic, Brookfield Clinic, Heaton Norris Clinic. *Warrington:* Derby Road Clinic. *Wigan:* Beech Hill Health Centre, College Road Clinic, Grasmere Street Health Centre, Haig Road Clinic, Liverpool Road Health Centre, Longshoot Lane Health Centre, Orrell Road Clinic, Pemberton Clinic, Platt Bridge Clinic, Queens Road Clinic, Standish Clinic
